# Supplementary figures and images for: Affinity-tagged SMAD1 and SMAD5 mouse lines reveal transcriptional reprogramming mechanisms during early pregnancy
Source: eLife. 2024 Mar 27;12:RP91434. doi: 10.7554/eLife.91434 (PMC10972565; doi:10.7554/eLife.91434)

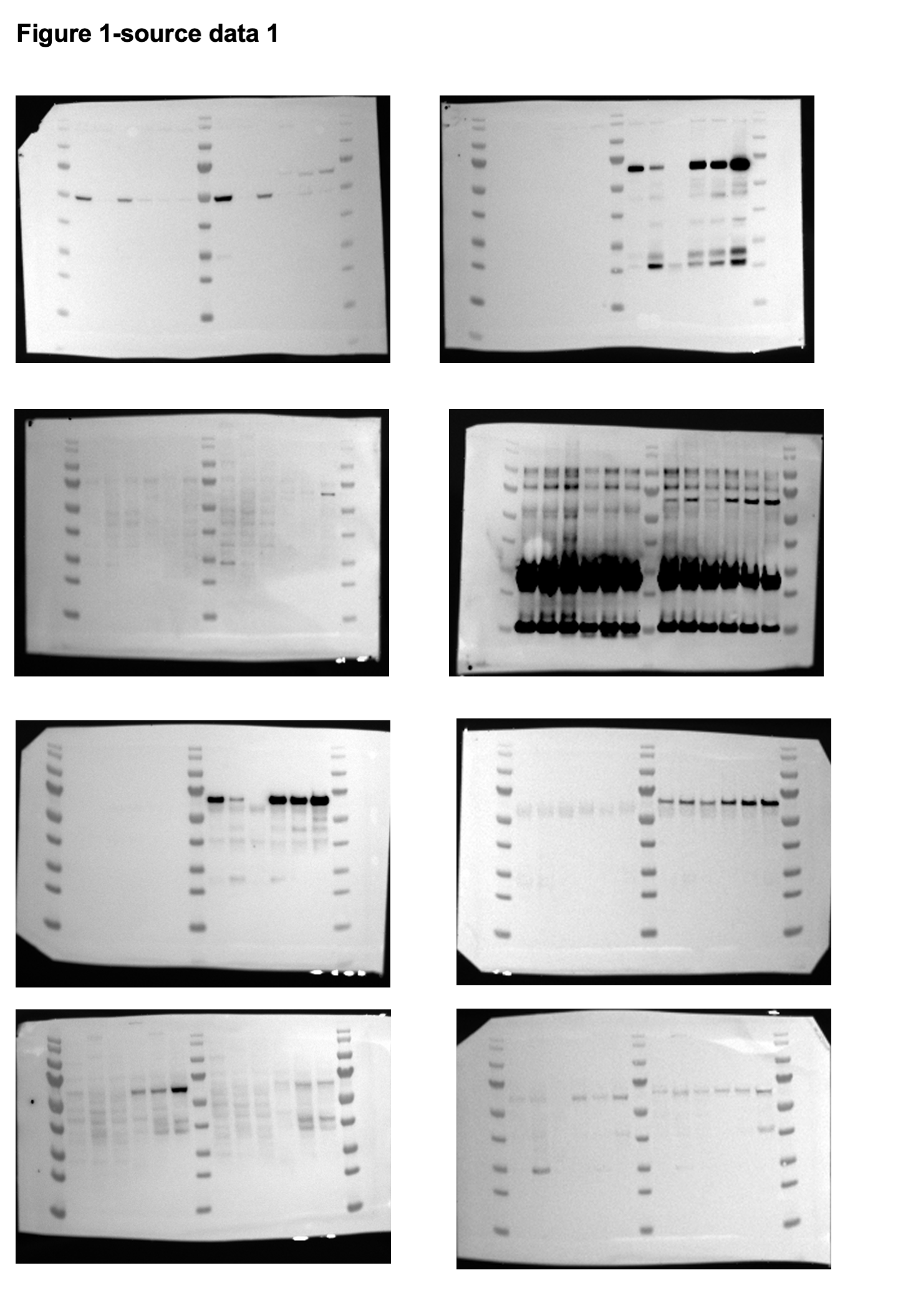

Supplement: Figure 1—source data 1. [file elife-91434-fig1-data1.tiff]

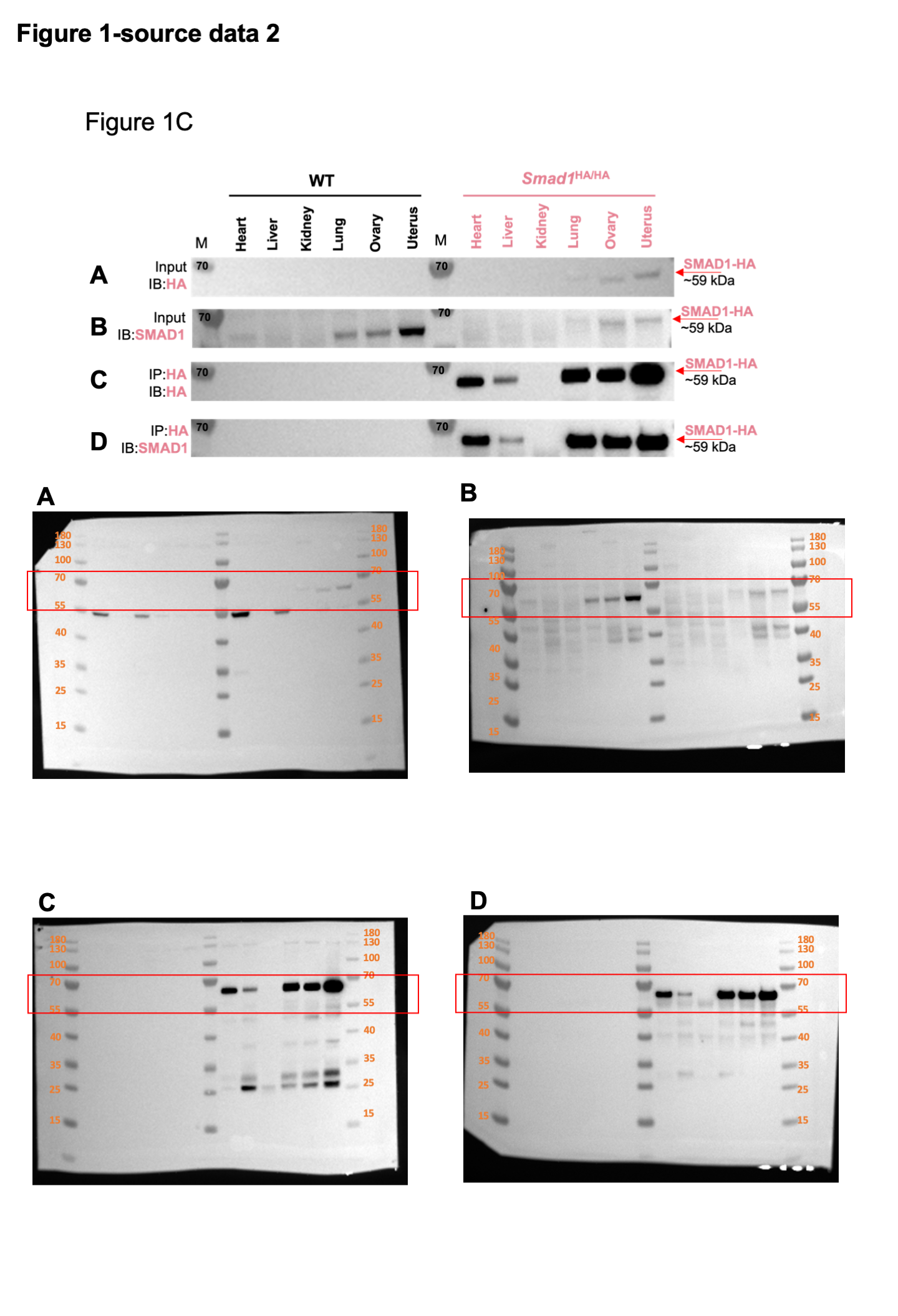

Supplement: Figure 1—source data 2. [file elife-91434-fig1-data2.tiff]

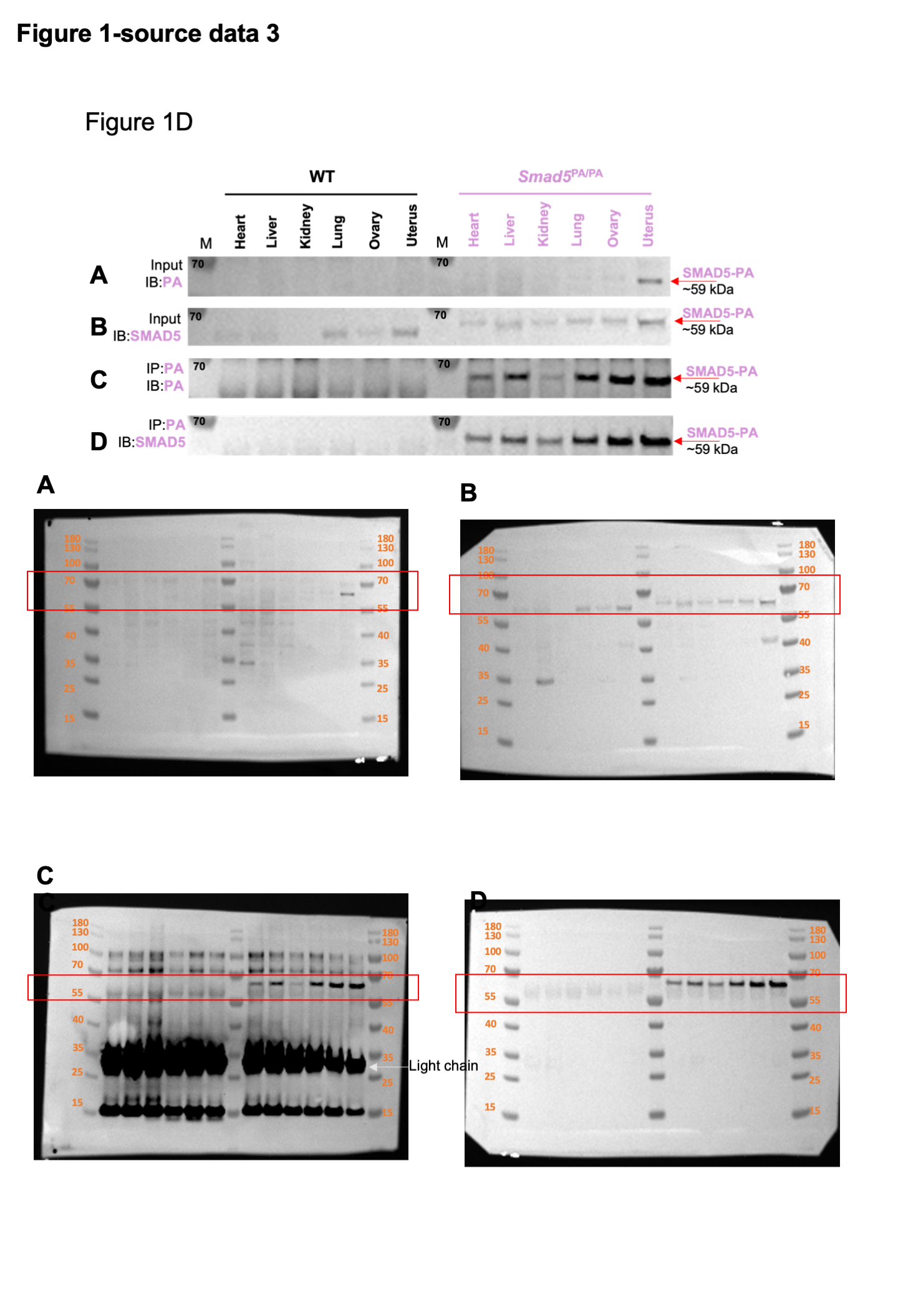

Supplement: Figure 1—source data 3. [file elife-91434-fig1-data3.tiff]
